# Perception of Medical Students on Anaesthesiology as a Specialty: A Systematic Review

**DOI:** 10.7759/cureus.73213

**Published:** 2024-11-07

**Authors:** Collins C Okeke, Adetolani P Gboyega-Adejuwon, Abdulahi Zubair, Promise U Okereke, Obinna E Ikegwuonu, Ebuka L Anyamene, Malipeh-Unim Undie, Love E Imoukhuede, Temiloluwa S Ojubolamo, Esther C Obiyo, Christian O Igibah, Raphael T Osele, Mazpa Ejikem

**Affiliations:** 1 Anaesthesiology, Surgery Interest Group of Africa, Lagos, NGA; 2 Dentistry, Surgery Interest Group of Africa, Lagos, NGA; 3 Anaesthesiology, Federal Teaching Hospital, Lokoja, NGA; 4 Anaesthesiology, State University of New York (SUNY) Downstate Health Sciences University, New York, USA

**Keywords:** anaesthesia, anaesthesiology, medical student, perception, specialty

## Abstract

Anaesthesia is integral to a wide range of clinical procedures; however, there is a global challenge in the anaesthesia workforce due to several contributing factors. This study seeks to assess the knowledge and perceptions of medical students regarding anaesthesia as a specialty. Understanding these perspectives is essential to addressing the global shortage of anaesthesia professionals. PubMed, Scopus, African Journal Online, and ProQuest were searched from the database inception to July 2024 to identify relevant studies. A total of 2,575 articles were identified. After title and abstract screening, 2,137 articles were excluded. After full-text screening, 2,098 articles were excluded. Eight articles were included in this qualitative analysis. We included primary studies published in peer-reviewed journals that reported the perception and choice of anaesthesiology as a future career by medical students in their clinical years (years five and six), with reasons, irrespective of medical institution, age, or gender, were included. In total, 2,135 students from six countries were included in the eight studies: Pakistan, Saudi Arabia, Nigeria, Austria, the United States of America, and South Africa, with Nigeria and Saudi Arabia each contributing two studies. The study designs utilised included cross-sectional surveys, descriptive cross-sectional studies, and questionnaire-based studies. The mean age of the students ranged from 23 to 28 years across the studies, with 748 male students, 530 female students, and 857 students whose gender was not specified. A significant proportion of students were aware of the role of anaesthetists; however, only 178 students expressed an interest in specialising in anaesthesia, with the most common reason being a genuine interest in the specialty. The studies reviewed identified several reasons why students did not consider anaesthesiology as a preferred speciality, including limited patient contact, which affects the doctor-patient relationship, lack of recognition or respect from peers, low income, limited availability of local job opportunities or training positions, and the presence of anaesthetic nurses. A critical factor highlighted across all studies was the length of exposure to anaesthesia during clinical rotations. Medical students recognise the importance of anaesthetists but are often deterred from pursuing the specialty due to various factors, which could be mitigated through enhanced exposure, increased visibility, financial incentives, and mentorship.

## Introduction and background

Anaesthesia is a specialty focused on managing patients undergoing surgical or diagnostic procedures through analgesia, amnesia, hypnosis, and muscle relaxation tailored to the procedure [1]. Techniques include general anaesthesia for full sedation, regional anaesthesia for targeted numbness with awareness, and monitored anaesthesia care for vital sign modulation during procedures [2]. Beyond its primary role, anaesthesia also covers pain management, and in some countries, intensive care medicine is integrated as a subspecialty or core component of anaesthesia training [3,4]. A study by Wacker and Staender highlighted the crucial role of anaesthesia within healthcare [5]. A 2015-2016 survey by the World Federation of Societies of Anaesthesiologists, spanning 153 countries, showed substantial disparities in physician anaesthesia provider (PAP) density between WHO regions and income groups, with 77 countries reporting <5 PAPs per 100,000 people, especially in African and Southeast Asian regions. Non-PAPs (NPAPs) are vital in low-resource areas [6]. However, challenges in recruiting and training a sufficient workforce persist globally, with studies exploring contributing factors [7-9]. Studies by Khan et al. [10], Field and Lennox [11], Emmanouil et al. [12], and Faponle [13] examine how medical students’ knowledge and perceptions influence their career choice in anaesthesia [10-13].

Several studies assess medical students’ awareness and attitudes toward anaesthesia. In Canada, graduating students displayed solid knowledge and positive perspectives on anaesthesia shaped by exposure [14]. In Saudi Arabia, while 92.9% of students had good knowledge, 83.1% had poor perceptions [2]. In the UK, factors influencing anaesthesia choice shifted over time, with lifestyle considerations like work hours becoming more impactful [12]. Single-centre studies in Pakistan and Nigeria showed that clinical clerkships improve perceptions, reducing misconceptions about anaesthetists’ roles. In Pakistan, awareness rose to 86%, though 34% retained negative views. In Nigeria, most students recognised anaesthetists’ critical roles (68.2%), but some saw their role as limited to surgery or undervalued compared to anaesthetic nurses [10,15].

Challenges within anaesthesiology may be discouraging, unpredictable patient responses, especially in dosing and pain management, misconceptions about the specialty’s scope, and limited exposure to different career options. A 2015 Indian study found that 31.6% of students were unaware of anaesthesia’s full scope during postgraduate counselling [16-18]. Globally, 5 billion people lack access to safe anaesthesia, partly due to low interest and awareness among students [10,6]. By improving exposure, mentorship, curriculum integration, and awareness of career opportunities, targeted interventions can enhance students’ understanding and enthusiasm, strengthening the future anaesthesia workforce [2,19-21].

This review aims to evaluate the awareness of medical students of the role of anaesthetists, their interest in specialising, and the push factors contributing to their lack of interest in this specialty.

## Review

Methods

This systematic review was conducted in accordance with the Preferred Reporting Items for Systematic Reviews and Meta-Analyses (PRISMA) extension for systematic reviews [22]. The study protocol was registered with PROSPERO (CRD42024568021).

*Inclusion Criteria* 

Primary studies published in peer-reviewed journals that reported the perception and choice of anaesthesiology as a future career by medical students in their clinical years (years five and six), with reasons, irrespective of medical institution, age, or gender, were included.

*Exclusion Criteria* 

Studies conducted outside of a medical institution or involving preclinical medical students or non-medical students were excluded. Additionally, study designs such as case reports, audits, opinions, reviews, meta-analyses, comments, and editorials were excluded.

Prevention is also very important in anaesthesiology and critical care, e.g., drug errors, cardiac arrest, etc. Monitoring and surveillance are other noteworthy attributes relevant to both streams. Vaccines are among the most cost-effective public health measures of disease control, whereas anaesthesiologists are trained to tackle adverse effects following immunisation (AEFI) as part of their curriculum.

A comprehensive search was performed from inception to July 7, 2024, using the PubMed, Scopus, African Journals Online (AJOL), and ProQuest databases. The keywords used were (((Perception) AND (Medical Student)) AND (Anaesthesia)), “Perception,” “Medical Student,” and “Anaesthesia.” The search details are provided in the Appendix (Table 4).

Duplication, title, and abstract screening were performed by five independent reviewers (C.C.O, E.L.A, L.E.I, E.C.O, and O.E.I) using the Rayyan systematic review software, according to predefined eligibility criteria. Potentially eligible studies were then screened for full-text review. Disagreements among reviewers were discussed, and if unresolved, an additional reviewer (A.Z.) was consulted.

Data were extracted from the articles related to the author, study year, sample size, mean age, gender, awareness of the role of anaesthetists, interest in anaesthesia, reasons for indicating interest in anaesthesia, and reasons for not being interested in anaesthesia.

The risk of bias in the included studies was assessed using the AXIS critical appraisal tool for cross-sectional studies provided in the Appendix (Table 5) [23]. This tool appraises study design quality, sample size, characteristics, measures, internal consistency, results, analysis, and limitations. Its checklist comprises 20 questions in three primary categories: quality of reporting (seven questions), study design quality (seven questions), and possible introduction of biases (six questions). Although it does not provide an established rule for determining the quality of each study, a percentage value was predetermined at the beginning of this study to identify publications as high or low quality. Publications with total appraisal scores equal to or exceeding 70% (i.e., at least 14 out of 20 questions scored as 1 or a score ≥ 14) were considered high quality, while scores between 60% and 69.9% were considered fair quality. Publications with scores below 60% were considered low quality [24]. 

Results

**Figure 1:**
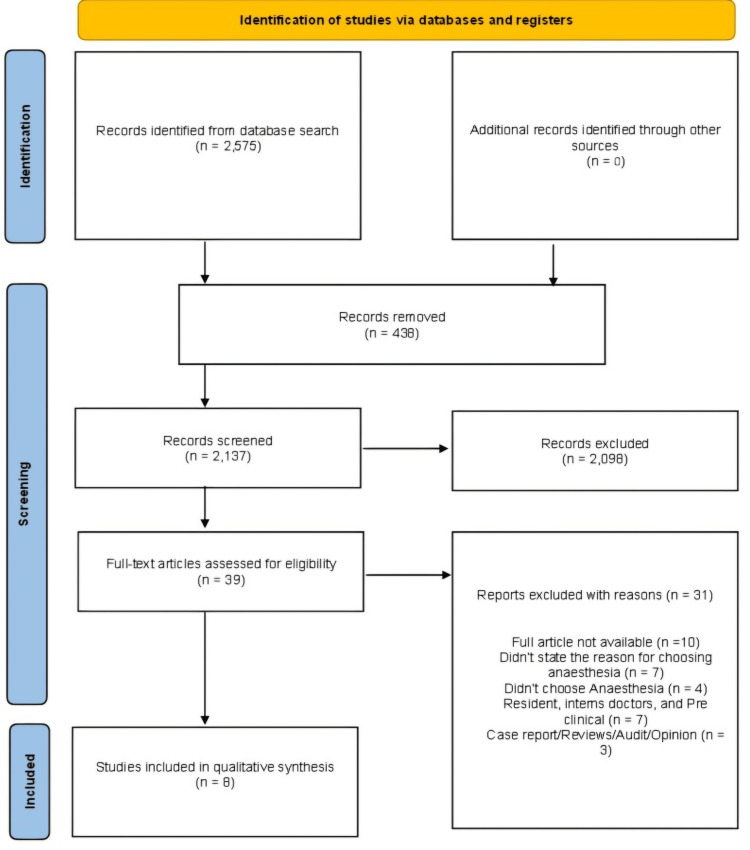
PRISMA flow diagram PRISMA, Preferred Reporting Items for Systematic Reviews and Meta-Analyses

Our search returned 2,575 articles, of which 2,137 were screened by title and abstract after duplicates were removed. Following the title and abstract screening, 2,098 articles were excluded, and 39 articles were subjected to full-text screening to determine their eligibility based on our inclusion criteria. Ultimately, eight articles were included in the final qualitative synthesis. Figure 1 displays the PRISMA flow diagram. Exclusions were made for various reasons, including failure to meet our inclusion criteria, unavailability of full articles, focus on populations outside our scope (such as resident doctors, interns, and preclinical medical students), and studies that did not specifically address the choice of anaesthesia as a specialty. Data screening and extraction were independently conducted by five authors, with a sixth reviewer consulted in cases of disagreement.

**Figure 2:**
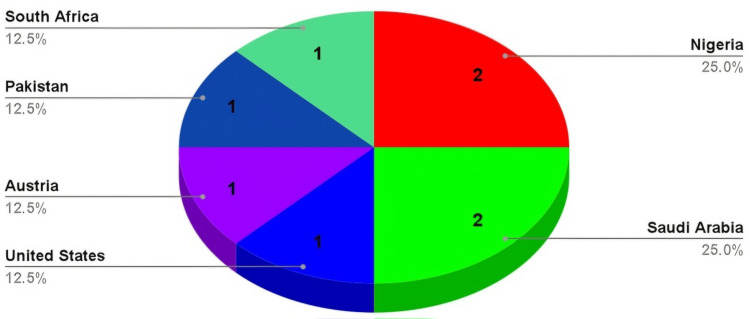
Study Locations Nigeria and Saudi Arabia contributed two studies each, while South Africa, Pakistan, Austria, and the United States of America contributed one article each.

In total, 2,135 students were included in eight studies from six countries (Saudi Arabia, Pakistan, Nigeria, South Africa, the United States of America, and Austria) [2,10,15,25-29], with Nigeria and Saudi Arabia contributing two studies each. Figure 2 displays the study location. The study period ranged from 1973 to 2024. The mean age of the reported students ranged from 23 to 28 years across the studies, although some studies did not report mean ages [2,10,26,28]. There were 748 male students and 530 female students, while the gender of 857 students was not specified.

Study Types 

The studies included in this review focused on final-year students and students in their clinical rotations who are interested in specialisation training and inquired about their interests in anaesthesia. The study types employed included cross-sectional surveys [10], descriptive cross-sectional studies [2], and questionnaire-based studies [15,25-29]. Table 1 summarises the study characteristics.

**Table 1:** Study characteristics

Author	Year	Country	Sample size	Mean age	Male	Female
Alrajban et al. [2]	2024	Saudi Arabia	379	NA	184	195
Khan et al. [10]	2011	Pakistan	150	NA	78	70
Nwasor [15]	2010	Nigeria	88	28	71	17
Gqiba et al. [25]	2017	South Africa	112	23	38	74
AlKhilaiwi et al. [26]	2018	Saudi Arabia	236	NA	153	82
Adeleye and Eze [27]	2010	Nigeria	296	25.85	216	80
Bruhn et al. [28]	1973	America	688	NA	0	0
Fitzgerald et al. [29]	2021	Austria	186	23	8	12

*Awareness of the Role of Anaesthetists* 

In the studies reviewed, a significant proportion of students were aware of the role of anaesthetists. Alrajban et al. (2024) [2] showed that 92% of students sampled knew the role of anaesthetists through their validated questionnaire, while Khan et al. (2011) [10] and Nwasor (2010) [15] also had similar findings in their studies. Some of the roles played by anaesthetists, as described in these studies, include a “vital role in surgery,” pre-and post-operative care of patients, roles in the emergency department and intensive care unit, pain management, and “assisting the surgeon.” 

*Specialty Interest* 

Only 178 students across the studies reviewed were interested in specialising in anaesthesia, with the most common reasons being an interest in the specialty [15], shorter training time [15], being mentally challenging [25], being afforded good working hours [25], a good lifestyle [26], potentially high income [27], and a wide array of opportunities within anaesthesia [29]. Students with exposure to anaesthesiology as part of their clinical rotation also pointed out that this experience inspired them to consider a career in anaesthesia [26]. Table 2 shows the various reasons for wanting to specialize in anaesthesia.

**Table 2:** Reasons for wanting to specialise in anaesthesia

Author	Reasons for wanting to specialise in anaesthesia
Alrajban et al. [2]	N/A
Khan et al. [10]	N/A
Nwasor [15]	Liked the practice of anaesthesia, felt that anaesthesia was interesting, and felt that it took a shorter time to train to become an anaesthetist
Gqiba et al. [25]	Interesting, mentally challenging, and afforded good working hours
AlKhilaiwi et al. [26]	Lifestyle, pressure from family and peers, patient care aspects, basic science/research aspects, and income
Adeleye et al. [27]	Potential for high income, availability of spare time, and opportunity for foreign jobs
Bruhn et al. [28]	Anesthesiology preceptorship and liked electives programs
Fitzgerald et al. [29]	Practical work, the wide spectrum of specialties, the care of patients in emergencies, the "high-tech” working field, the high number of available positions, and interdisciplinary work, especially in the operation room

Push Factors

Students sampled in the studies reviewed provided several reasons for not considering anaesthesiology as a potential specialty. These included minimal contact with patients, which affects the doctor-patient relationship [2,10,15], lack of recognition or respect from peers [2,10], low income [2], lack of available jobs locally [15] or training positions [26,29], and the presence of anaesthetic nurses [15]. A significant factor across all the studies sampled was the limited exposure to anaesthesia during clinical rotations [10,15,27,29]. Table 3 lists some of the reasons for the lack of interest in anaesthesia.

**Table 3:** Reasons for lack of interest in anaesthesia

Author	Reasons for not wanting to specialise in anaesthesia
Alrajban et al. [2]	Controllable lifestyle (ability to control work hours), presence of doctor-patient relationship, and income Prestige of specialty
Khan et al. [10]	Pre-clerkship: minimum patient contact, no exposure to specialty, lack of recognition by patients, and lack of recognition by peers
	Post-clerkship: minimum patient contact, no exposure to specialty, lack of recognition by patients, lack of recognition by peers
Nwasor [15]	Short duration of exposure in medical school, presence of anaesthetic nurses, they work behind the scenes, no financial Inducement, lack of jobs In Nigeria, and others
Gqiba et al. [25]	Boring, stressful, and frightening because it is more exciting to be hands-on than in the background; anaesthetists are painted as helpers or assistants, and there’s no respect for it
AlKhilaiwi et al. [26]	Fear of not matching into the field and no exposure to anaesthesia rotation in medical school
Adeleye et al. [27]	Short duration of exposure in medical school and inadequate information
Bruhn et al. [28]	Unfavourable clinical experience while in medical school and routine exposure to anesthesiology
Fitzgerald et al. [29]	Lack of knowledge about possibilities of work & career in the specialty, lack of contact with the specialty during the university training, lack of training positions dependence of work and career in a hospital, and lack of communication and follow-up with patients

Discussion 

This systematic review presents insightful data on medical students’ awareness of the role of anaesthetists, their interest in specialising in anaesthesia, and the push factors contributing to their lack of interest in this specialty. The analysis encompasses a wide temporal range and diverse geographic locations, reflecting varied educational and healthcare contexts.

Awareness of the Role of Anaesthetists

The review indicates a high level of awareness among medical students regarding the role of anaesthetists. For instance, Alrajban et al. (2024) [2] reported that 92% of the sampled students understood the critical functions anaesthetists perform, such as involvement in surgery, pre- and post-operative care, emergency care, and pain management. Similar findings were reported by Khan et al. (2011) [10] and Nwasor (2010) [15], suggesting that students recognise the multifaceted responsibilities of anaesthetists in patient care. This awareness is crucial as it forms the foundation for informed career choices among medical students. Recognising anaesthetists’ roles beyond the operating theatre, including their contributions to intensive care units and emergency departments, highlights the specialty’s broad scope. This broad understanding is vital for attracting students to the field, as it dispels the notion that anaesthetists are mere “assistants to surgeons” and emphasises their independent and critical role in patient management. For example, in Saudi Arabia, over 90% of medical students understood the role of anaesthetists, recognizing responsibilities such as surgical support, pain management, and critical care [30]. Khan and Ahmad (2023) believe that anaesthesiologists can play an important role at the community level by forming a synergy with the community medicine doctors, as they are among the best persons to implement triage and maximize the outcome in terms of reducing mortality, morbidity, and disability [31]. The COVID-19 pandemic showcased the importance of anaesthetics as prevention is also very important in anaesthesiology and critical care, e.g., drug errors, cardiac arrest, etc. Monitoring and surveillance are other noteworthy attributes relevant to both streams. Vaccines are among the most cost-effective public health measures of disease control, whereas anaesthesiologists are trained to tackle AEFI as part of their curriculum. They are also involved in the planning of health management and policy-making [31].

Interest in Specialising in Anaesthesia

Despite the high awareness, interest in specializing in anaesthesia remains relatively low, with only 178 (8%) students expressing such interest across the studies. The motivations for choosing this specialty are diverse. For instance, students are attracted by the specialty’s intellectually stimulating nature [15,25], the prospect of good working hours [25], and a favourable lifestyle [26]. The potential for high income and a wide array of career opportunities within anaesthesia also play significant roles [27,29]. Clinical exposure to anaesthesia appears to significantly influence students’ interest. AlKhilaiwi et al. (2018) [26] and Bruhn et al. (1973) [28] highlighted that hands-on experience and elective programs in anaesthesia during medical school positively impacted students’ career choices, underscoring the importance of incorporating comprehensive anaesthesia rotations in medical curricula. According to Tyagi et al. (2012), the choice of anaesthesiology was influenced greatly by the opportunity to do procedures and diverse clinical spectrum, higher income earning potential, and intellectual stimulation or challenge [32]. Turner et al. (2005) highlighted good work hours, working conditions, and diverse job experience as influential factors in choosing anaesthesia after graduation [33].

Push Factors Against Specialising in Anaesthesia

Several factors dissuade students from pursuing a career in anaesthesia. A recurring theme is the perceived lack of patient contact, which affects the doctor-patient relationship [10,11,15]. This perception could stem from limited exposure to the specialty during medical training, which many students noted as insufficient [10,27,29]. Additionally, the specialty’s lack of visibility and recognition among peers and patients contributes to its unattractiveness [10,15]. Poor economic factors also play a role in some countries, leading to the inability of some countries to pay the anaesthesiology well to meet the rising inflation in the economy, with some students citing low income and limited job opportunities locally as deterrents [15]. Furthermore, the presence of anaesthetic nurses and the perception of anaesthetists working “behind the scenes” diminish the specialty’s appeal [15]. Tyagi et al. (2012) also highlighted the lack of doctor-to-patient interaction and awareness in society as the major push factors [32].

Potential Impact on Clinical Practice

A report by the Royal College of Anaesthetic revealed a shortfall of 11,000 anaesthetic staff by 2040, which would prevent 8.25 million operations from taking place per year. Currently, there is a shortage of anaesthetic and its preventing, and it is preventing patients from getting the operations they need [34]. A workshop survey carried out by Hu and Jiang emphasized the lack of trained anaesthesia professionals is one of the most important barriers to essential anaesthesia care [35]. The shortage of anaesthesia workforce can also lead to staff burnout, which has been a widely studied factor linked to poorer quality of life for anaesthesia professionals and reduced quality of care for patients [35]. 

The reluctance of medical students to pursue anaesthesiology has implications for healthcare delivery, particularly in regions with an existing anaesthesiologist shortage. Studies indicate that low-density anaesthesia providers, especially in parts of Africa and Southeast Asia where provider density is less than five per 100,000 population, lead to gaps in perioperative care and reduced access to essential pain management services [31,36]. Addressing these shortages by enhancing interest in anaesthesiology could substantially improve surgical safety and pain management in low-resource settings. For example, the WHO has identified insufficient access to anaesthetic care as a factor in increased perioperative mortality, a gap that could be alleviated by a more robust anaesthesia workforce [31].

Future research should prioritize interventions to enhance interest in anaesthesiology, such as incorporating longer anaesthesia rotations in medical curricula and implementing mentorship programs. Longitudinal studies measuring the impact of clinical rotations on career selection could further elucidate how educational experiences shape specialty interests. Additionally, investigating socio-economic influences on specialty choice, such as salary expectations, job stability, and perceived job satisfaction, would provide critical insights, particularly in regions where low income and limited job opportunities deter students from choosing anaesthesiology [31,36].

Exploring interprofessional dynamics might also shed light on how the perceived prestige of anaesthesiologists, among other specialists, affects students’ interest. Some studies suggest that students avoid fields where professional recognition appears diminished, indicating that enhancing anaesthesiology’s visibility and prestige could play a role in attracting more students.

Recommendations

Several strategies could be implemented to enhance interest in anaesthesia. First, extending the duration and quality of anaesthesia rotations in medical school by at least four weeks can provide students with a deeper understanding and appreciation of the specialty. Tyagi et al. suggest an early introduction of the subject into the undergraduate curriculum [32]. Turner et al. observed that after the inclusion of anaesthesia, resuscitation, and intensive therapy in the undergraduate curriculum, the percentage of fresh graduates opting for an anaesthesiology career increased from 4.6% to 11.5% [33]. Interactive and hands-on experiences can demystify the role of anaesthetists and highlight the intellectual challenges and rewards of the field. Efforts to raise the profile of anaesthetists within the medical community and among patients can help mitigate the lack of recognition. By highlighting the critical role anaesthetists play in patient care across various settings, the specialty’s status can be elevated. Addressing concerns about income and job opportunities through financial incentives and showcasing the diverse career paths available within anaesthesia can also attract more students to the specialty. Additionally, establishing mentorship programs where experienced anaesthetists guide and support medical students can foster interest and provide valuable career insights. Addressing the importance of increasing pay and recognition is one of the pillars of making anaesthesia appealing to medical students. This will give job satisfaction to practising anaesthesiologists.

Khan and Karim suggest continuing medical education (CME) opportunities should be made more accessible to medical students [36]. Governments and healthcare institutions should organize regular CME workshops, conferences, and online courses. Cooperation with international anaesthesia organizations can also facilitate knowledge sharing and skill development. Addressing the issue of low remuneration, governments and private healthcare institutions should consider revising payment structures to ensure fair and competitive service remuneration. Furthermore, introducing performance-based incentives, professional development allowances, and improved working conditions can also enhance job satisfaction and motivate anaesthesiologists to provide unhindered services. Collaboration with healthcare administrators and surgical consultants can help bridge communication gaps and empower anaesthesiologists to play a more significant role in patient care planning [36].

Study strengths and limitations 

The review process was thorough, with a large initial pool of articles (n = 2,575) being narrowed down to eight studies through rigorous screening. This meticulous approach enhances the credibility of the findings. Data screening and extraction were conducted independently by five authors, with a sixth reviewer resolving any disagreements, ensuring a robust and unbiased synthesis of the available evidence. However, several high-quality articles that met the inclusion criteria were not available for full-text screening and data extraction, hence limiting the number of articles for analysis. There is a possibility of publication bias as the researcher might not get the desired outcome and then end up not publishing the paper. Additionally, some studies that aligned with the scope of the review did not explicitly express the reasons guiding medical students’ choices for or against anaesthesia as a specialty.

This systematic review also highlights gaps and inconsistencies in the literature. Geographic representation was limited, with most high-quality studies originating from North America and Europe and fewer from Africa and Asia. Variability in exposure to anaesthesiology during medical training also poses a challenge when comparing findings globally. To obtain a more comprehensive picture, future studies should prioritise underrepresented regions and provide standardised metrics for assessing exposure and interest across medical curricula.

## Conclusions

While medical students are generally aware of the vital role anaesthetists play in surgical and overall patient care, interest in the specialty remains limited due to several push factors. Addressing these concerns through enhanced exposure, increased visibility, economic incentives, and mentorship could significantly improve the appeal of anaesthesia as a career choice for potential anaesthetists. This systematic review reveals that while medical students generally have a high awareness of the critical roles that anaesthetists play in surgical care, this awareness does not strongly translate into a desire to specialise in the field. Only a small fraction of students expressed interest in anaesthesiology, motivated by factors such as work-life balance, income potential, and intellectual challenge. However, many are deterred by the perceived lack of patient contact, lower professional recognition, limited exposure during medical training, and concerns about job availability. These findings suggest a need for enhanced exposure to anaesthesiology, better representation of its importance, and addressing misconceptions to improve their understanding of the speciality and hence increase their interest in this important specialty.

## Appendices

Author contribution

Collins C. Okeke (project administration, abstract, introduction, methods, results, and discussion), Adetolani P. Gboyega-Adejuwon ( abstract, introduction, and results), Abdulahi Zubair (abstract and results), Promise U. Okereke (introduction, discussion, and conclusion), Obinna E. Ikegwuonu (abstract and methods), Ebuka L. Anyamene (methods and results), Malipeh-Unim Undie (abstract and introduction), Love E. Imoukhuede (abstract and methods), Temiloluwa S. Ojubolamo (abstract and methods), Esther C. Obiyo (abstract and methods), Christian O. Igibah (abstract and discussion), Raphael T. Osele (reviewer), and Mazpa Ejikem (reviewer)

**Table 4:** Search strategy

Search strategy	Database	No. of article
(((Perception) AND (Medical Student)) AND (Anaesthesia))	Pubmed	183
((Medical Student) AND (Anaesthesia)) AND (Specialty)	Pubmed	1655
"Perception" "Medical Student" "Anesthesia"	Scopus	82
"Medical Student" "Anaesthesia" "Specialty"	Scopus	184
"Perception" "Medical Student" "Anesthesia"	Ajol	23
"Perception" "Medical Student" "Anesthesia"	Proquest	507

**Table 5:** AXIS critical appraisal tool for cross-sectional studies

S/N	Question	Yes	No	Don’t know/comment
Title: Medical students’ knowledge and perception of anaesthesia: an insight into anesthesiology as a career choice				
Author: Alrajban et al. [2]				
Introduction				
1	Were the aims/objectives of the study clear?	Yes		
Methods				
2	Was the study design appropriate for the stated aim(s)?	Yes		
3	Was the sample size justified?	Yes		
4	Was the target/reference population clearly defined? (Is it clear who the research was about?)	Yes		
5	Was the sample frame taken from an appropriate population base so that it closely represented the target/reference population under investigation?	Yes		
6	Was the selection process likely to select subjects/participants that were representative of the target/reference population under investigation?	Yes		
7	Were measures undertaken to address and categorise non-responders?	Yes		
8	Were the risk factors and outcome variables measured appropriately to the aims of the study?	Yes		
9	Were the risk factor and outcome variables measured correctly using instruments/measurements that had been trialled, piloted or published previously?	Yes		
10	Is it clear what was used to determine statistical significance and/or precision estimates? (e.g. p-values and confidence intervals)	Yes		
11	Were the methods (including statistical methods) sufficiently described to enable them to be repeated?	Yes		
Results				
12	Were the basic data adequately described?	Yes		
13	Does the response rate raise concerns about non-response bias?		No	
14	If appropriate, was information about non-responders described?		No	
15	Were the results internally consistent?	Yes		
16	Were the results presented for all the analyses described in the methods?	Yes		
Discussion				
17	Were the authors' discussions and conclusions justified by the results?	Yes		
18	Were the limitations of the study discussed?	Yes		
	Others			
19	Were there any funding sources or conflicts of interest that may affect the authors’ interpretation of the results?		No	
20	Was ethical approval or consent of participants attained?	Yes		
Score: 17				
Title: Anaesthesia as a career choice in a developing country; effect of clinical clerkship				
Author: Khan et al. [10]				
Introduction				
1	Were the aims/objectives of the study clear?	Yes		
Methods				
2	Was the study design appropriate for the stated aim(s)?	Yes		
3	Was the sample size justified?	Yes		
4	Was the target/reference population clearly defined? (Is it clear who the research was about?)	Yes		
5	Was the sample frame taken from an appropriate population base so that it closely represented the target/reference population under investigation?	Yes		
6	Was the selection process likely to select subjects/participants that were representative of the target/reference population under investigation?	Yes		
7	Were measures undertaken to address and categorise non-responders?	Yes		
8	Were the risk factors and outcome variables measured appropriately to the aims of the study?			Don’t know
9	Were the risk factor and outcome variables measured correctly using instruments/measurements that had been trialled, piloted or published previously?	Yes		
10	Is it clear what was used to determine statistical significance and/or precision estimates? (e.g. p-values and confidence intervals)	Yes		
11	Were the methods (including statistical methods) sufficiently described to enable them to be repeated?	Yes		
Results				
12	Were the basic data adequately described?	Yes		
13	Does the response rate raise concerns about non-response bias?	Yes		
14	If appropriate, was information about non-responders described?		No	
15	Were the results internally consistent?		No	
16	Were the results presented for all the analyses described in the methods?	Yes		
Discussion				
17	Were the authors' discussions and conclusions justified by the results?	Yes		
18	Were the limitations of the study discussed?	Yes		
Others				
19	Were there any funding sources or conflicts of interest that may affect the authors’ interpretation of the results?			
20	Was ethical approval or consent of participants attained?	Yes		
Score: 16				
Title: Perception of final-year medical students about choice of anaesthesia as a specialty				
Author: Nwasor [15]				
Introduction				
1	Were the aims/objectives of the study clear?	Yes		
Methods				
2	Was the study design appropriate for the stated aim(s)?	Yes		
3	Was the sample size justified?	Yes		
4	Was the target/reference population clearly defined? (Is it clear who the research was about?)	Yes		
5	Was the sample frame taken from an appropriate population base so that it closely represented the target/reference population under investigation?	Yes		
6	Was the selection process likely to select subjects/participants that were representative of the target/reference population under investigation?	Yes		
7	Were measures undertaken to address and categorise non-responders?	Yes		
8	Were the risk factors and outcome variables measured appropriately to the aims of the study?	yes		
9	Were the risk factor and outcome variables measured correctly using instruments/measurements that had been trialled, piloted or published previously?			Not specified
10	Is it clear what was used to determine statistical significance and/or precision estimates? (e.g. p-values and confidence intervals)		No	
11	Were the methods (including statistical methods) sufficiently described to enable them to be repeated?	Yes		
Results				
12	Were the basic data adequately described?	Yes		
13	Does the response rate raise concerns about non-response bias?	Yes		
14	If appropriate, was information about non-responders described?		No	
15	Were the results internally consistent?	Yes		
16	Were the results presented for all the analyses described in the methods?	Yes		
Discussion				
17	Were the authors' discussions and conclusions justified by the results?	Yes		
18	Were the limitations of the study discussed?	Yes		
Others				
19	Were there any funding sources or conflicts of interest that may affect the authors’ interpretation of the results?			Not specified
20	Was ethical approval or consent of participants attained?		No	
Score: 15				
Title: Perceptions of final-year UKZN medical students about anaesthesia as a specialty choice				
Author: Gqiba et al. [25]				
Introduction				
1	Were the aims/objectives of the study clear?	Yes		
Methods				
2	Was the study design appropriate for the stated aim(s)?	Yes		
3	Was the sample size justified?	Yes		
4	Was the target/reference population clearly defined? (Is it clear who the research was about?)	Yes		
5	Was the sample frame taken from an appropriate population base so that it closely represented the target/reference population under investigation?	Yes		
6	Was the selection process likely to select subjects/participants that were representative of the target/reference population under investigation?	Yes		
7	Were measures undertaken to address and categorise non-responders?	Yes		
8	Were the risk factors and outcome variables measured appropriately to the aims of the study?	Yes		
9	Were the risk factor and outcome variables measured correctly using instruments/measurements that had been trialled, piloted or published previously?	Yes		
10	Is it clear what was used to determine statistical significance and/or precision estimates? (e.g. p-values and confidence intervals)	Yes		
11	Were the methods (including statistical methods) sufficiently described to enable them to be repeated?	Yes		
Results				
12	Were the basic data adequately described?	Yes		
13	Does the response rate raise concerns about non-response bias?		No	
14	If appropriate, was information about non-responders described?		No	
15	Were the results internally consistent?		No	
16	Were the results presented for all the analyses described in the methods?	Yes		
Discussion				
17	Were the authors' discussions and conclusions justified by the results?	Yes		
18	Were the limitations of the study discussed?	Yes		
Others				
19	Were there any funding sources or conflicts of interest that may affect the authors’ interpretation of the results?			Not specified
20	Was ethical approval or consent of participants attained?	Yes		
Score: 16				
Title: Medical students’ attitude toward anesthesia as a future career				
Author: AlKhilaiwi et al. [26]				
Introduction				
1	Were the aims/objectives of the study clear?	Yes		
Methods				
2	Was the study design appropriate for the stated aim(s)?	Yes		
3	Was the sample size justified?	Yes		
4	Was the target/reference population clearly defined? (Is it clear who the research was about?)	Yes		
5	Was the sample frame taken from an appropriate population base so that it closely represented the target/reference population under investigation?	Yes		
6	Was the selection process likely to select subjects/participants that were representative of the target/reference population under investigation?	Yes		
7	Were measures undertaken to address and categorise non-responders?	Yes		
8	Were the risk factors and outcome variables measured appropriately to the aims of the study?		No	
9	Were the risk factor and outcome variables measured correctly using instruments/measurements that had been trialled, piloted or published previously?		No	
10	Is it clear what was used to determine statistical significance and/or precision estimates? (e.g. p-values and confidence intervals)	Yes		
11	Were the methods (including statistical methods) sufficiently described to enable them to be repeated?	Yes		
Results				
12	Were the basic data adequately described?	Yes		
13	Does the response rate raise concerns about non-response bias?		No	
14	If appropriate, was information about non-responders described?		No	
15	Were the results internally consistent?	Yes		
16	Were the results presented for all the analyses described in the methods?	Yes		
Discussion				
17	Were the authors' discussions and conclusions justified by the results?	Yes		
18	Were the limitations of the study discussed?		No	
Others				
19	Were there any funding sources or conflicts of interest that may affect the authors’ interpretation of the results?		No	
20	Was ethical approval or consent of participants attained?	Yes		
Score: 14				
Title: Anticipated specialties and influencing factors among final year medical students in a Nigerian university				
Author: Adeleye et al. [27]				
Introduction				
1	Were the aims/objectives of the study clear?	Yes		
Methods				
2	Was the study design appropriate for the stated aim(s)?	Yes		
3	Was the sample size justified?	Yes		
4	Was the target/reference population clearly defined? (Is it clear who the research was about?)	Yes		
5	Was the sample frame taken from an appropriate population base so that it closely represented the target/reference population under investigation?	Yes		
6	Was the selection process likely to select subjects/participants that were representative of the target/reference population under investigation?	Yes		
7	Were measures undertaken to address and categorise non-responders?		No	
8	Were the risk factors and outcome variables measured appropriately to the aims of the study?		No	
9	Were the risk factor and outcome variables measured correctly using instruments/measurements that had been trialled, piloted or published previously?		No	
10	Is it clear what was used to determine statistical significance and/or precision estimates? (e.g. p-values and confidence intervals)	Yes		
11	Were the methods (including statistical methods) sufficiently described to enable them to be repeated?	Yes		
Results				
12	Were the basic data adequately described?	Yes		
13	Does the response rate raise concerns about non-response bias?		No	
14	If appropriate, was information about non-responders described?		No	
15	Were the results internally consistent?	Yes		
16	Were the results presented for all the analyses described in the methods?	Yes		
Discussion				
17	Were the authors' discussions and conclusions justified by the results?	Yes		
18	Were the limitations of the study discussed?	Yes		
Others				
19	Were there any funding sources or conflicts of interest that may affect the authors’ interpretation of the results?			Not Specified
20	Was ethical approval or consent of participants attained?	Yes		
Score: 14				
Title: Senior medical students' knowledge of and attitudes toward anesthesiology in ten medical schools				
Author: Bruhn et al. [28]				
Introduction				
1	Were the aims/objectives of the study clear?	Yes		
Methods				
2	Was the study design appropriate for the stated aim(s)?	Yes		
3	Was the sample size justified?	Yes		
4	Was the target/reference population clearly defined? (Is it clear who the research was about?)	Yes		
5	Was the sample frame taken from an appropriate population base so that it closely represented the target/reference population under investigation?	Yes		
6	Was the selection process likely to select subjects/participants that were representative of the target/reference population under investigation?	Yes		
7	Were measures undertaken to address and categorise non-responders?	Yes		
8	Were the risk factors and outcome variables measured appropriately to the aims of the study?		No	
9	Were the risk factor and outcome variables measured correctly using instruments/measurements that had been trialled, piloted or published previously?		No	
10	Is it clear what was used to determine statistical significance and/or precision estimates? (e.g. p-values and confidence intervals)	Yes		
11	Were the methods (including statistical methods) sufficiently described to enable them to be repeated?	Yes		
Results				
12	Were the basic data adequately described?	Yes		
13	Does the response rate raise concerns about non-response bias?	Yes		
14	If appropriate, was information about non-responders described?		No	
15	Were the results internally consistent?	Yes		
16	Were the results presented for all the analyses described in the methods?	Yes		
Discussion				
17	Were the authors' discussions and conclusions justified by the results?	Yes		
18	Were the limitations of the study discussed?	Yes		
Others				
19	Were there any funding sources or conflicts of interest that may affect the authors’ interpretation of the results?			Not specified
20	Was ethical approval or consent of participants attained?	Yes		
Score: 16				
Title: Development, implementation, evaluation, and long-term outcome of a program to increase student interest in anesthesia and intensive care training				
Author: Fitzgerald et al. [29]				
Introduction				
1	Were the aims/objectives of the study clear?	Yes		
Methods				
2	Was the study design appropriate for the stated aim(s)?	Yes		
3	Was the sample size justified?	Yes		
4	Was the target/reference population clearly defined? (Is it clear who the research was about?)	Yes		
5	Was the sample frame taken from an appropriate population base so that it closely represented the target/reference population under investigation?	Yes		
6	Was the selection process likely to select subjects/participants that were representative of the target/reference population under investigation?	Yes		.
7	Were measures undertaken to address and categorise non-responders?			No
8	Were the risk factors and outcome variables measured appropriately to the aims of the study?		No	
9	Were the risk factor and outcome variables measured correctly using instruments/measurements that had been trialled, piloted or published previously?	Yes		
10	Is it clear what was used to determine statistical significance and/or precision estimates? (e.g. p-values and confidence intervals)		No	
11	Were the methods (including statistical methods) sufficiently described to enable them to be repeated?		No	
Results				
12	Were the basic data adequately described?	Yes		
13	Does the response rate raise concerns about non-response bias?	Yes		
14	If appropriate, was information about non-responders described?		No	
15	Were the results internally consistent?	Yes		
16	Were the results presented for all the analyses described in the methods?	Yes		
Discussion				
17	Were the authors' discussions and conclusions justified by the results?	Yes		
18	Were the limitations of the study discussed?		No	
Others				
19	Were there any funding sources or conflicts of interest that may affect the authors’ interpretation of the results?		No	
20	Was ethical approval or consent of participants attained?	Yes		
Score: 13				
